# Decoding signaling architectures: CAR versus TCR dynamics in solid tumor immunotherapy: CAR versus TCR signaling in immunotherapy

**DOI:** 10.3724/abbs.2025190

**Published:** 2025-10-23

**Authors:** Zui Chen, Xin Zhou

**Affiliations:** 1 School of Basic Medicine Capital Medical University Beijing 100069 China; 2 Institute for Immunology Chinese Institutes for Medical Research Beijing 100069 China

**Keywords:** TCR, CAR-T, solid tumor, immunotherapy

## Abstract

The T cell receptor (TCR) initiates signaling by specifically recognizing peptide-MHC complexes, triggering the phosphorylation of CD3 chain immunoreceptor tyrosine-based activation motifs (ITAMs). This recruits kinases such as ZAP70, triggering a tightly regulated signaling cascade that governs T cell activation, differentiation, and effector functions. In contrast, the chimeric antigen receptor (CAR) is a synthetic construct that bypasses MHC restriction by fusing an antigen-binding domain with intracellular signaling modules (usually CD3ζ and co-stimulatory domains) from the TCR complex and other receptors. CAR-T cell therapy has revolutionized the treatment of hematologic malignancies, resulting in durable remission of B-cell leukemia, lymphoma, and multiple myeloma. However, its efficacy in solid tumors is limited by intrinsic barriers: poor CAR-T-cell trafficking/infiltration into tumors, the immunosuppressive tumor microenvironment (TME), intratumoral metabolic competition, and tumor antigen heterogeneity/loss. To improve CAR-T-cell function in solid tumors, numerous studies have explored multiple strategies: engineering CARs to boost immune synapse formation via optimized receptor clustering, increasing the ITAM number/strength to amplify downstream signaling, and incorporating novel/multiple co-stimulatory domains to sustain T-cell activation and persistence. Additionally, approaches include the use of CAR-T cells that secrete pro-inflammatory cytokines, epigenetic reprogramming to preserve T-cell stemness and functionality, and the use of synthetic biology tools for tunable/logic-gated CAR activation. Here, we summarize the current understanding of CAR signaling dynamics and highlight recent breakthrough strategies designed to overcome these challenges in solid tumors. These advances narrow the liquid-solid tumor efficacy gap, holding promise for better clinical outcomes in patients with solid malignancies and a new era of personalized immunotherapy.

## Introduction

Immunotherapy has emerged as a transformative approach in oncology, with T cell-based therapies at the forefront of innovation. Among these, CAR-engineered T cells and TCR-based therapies represent two powerful strategies that harness the immune system to target malignancies. While CAR-T cells have demonstrated remarkable success in hematologic cancers by recognizing surface antigens independent of presentation, their efficacy in solid tumors remains limited due to challenges such as immunosuppressive microenvironments, poor trafficking, and antigen heterogeneity. In contrast, TCR-based therapies leverage the natural precision of MHC-restricted antigen recognition, offering potential against intracellular targets but facing hurdles related to MHC downregulation and off-target toxicity.

Understanding the fundamental differences in signaling architecture between CAR and TCR is critical to optimizing T cell therapies for solid tumors. This review delves into the structural and functional divergence between these two systems, examines the role of various co-stimulatory domains in shaping CAR-T cell fate, and surveys current clinical trials and innovative strategies aimed at overcoming the barriers to effective solid tumor immunotherapy. By integrating insights from basic science and clinical advances, we aim to highlight pathways toward more durable and targeted immunotherapeutic interventions.

## CAR versus TCR Signaling: Structural and Functional Divergence

### CAR signaling

Chimeric antigen receptor (CAR) is a type of artificial receptor designed to mimic the T-cell receptor (TCR) by incorporating intracellular signaling domains from the TCR complex. Their modular design comprises four modules: an antigen recognition domain, a hinge region, a transmembrane (TM) domain, and an intracellular signaling domain [ 1, 2] . The antigen recognition domain is often derived from the variable regions of a monoclonal antibody, such as a single-chain variable fragment (scFv), variable heavy domain (VH), or light chain, which can recognize cell-surface and soluble antigens [ 3– 5] . Non-antibody approaches, such as natural ligand/receptor pairs, are also used for the antigen recognition domain [ 6– 9] . The hinge region provides flexibility and distance for synapse formation and influences receptor accessibility, stability, and signaling [10]. The TM domain anchors the CAR to the cell membrane and plays a critical role in modulating its surface expression, stability, and propensity for receptor aggregation [11]. This extracellular domain is followed by a transmembrane region and intracellular signaling sequences from one (for second generation CARs) or two (for third generation CARs) co-stimulatory receptors, followed by the intracellular region of CD3ζ [12]. This enables CAR-T cells to recognize and destroy cancer cells like how native T cells recognize and eliminate infected cells.

**Figure 1:**
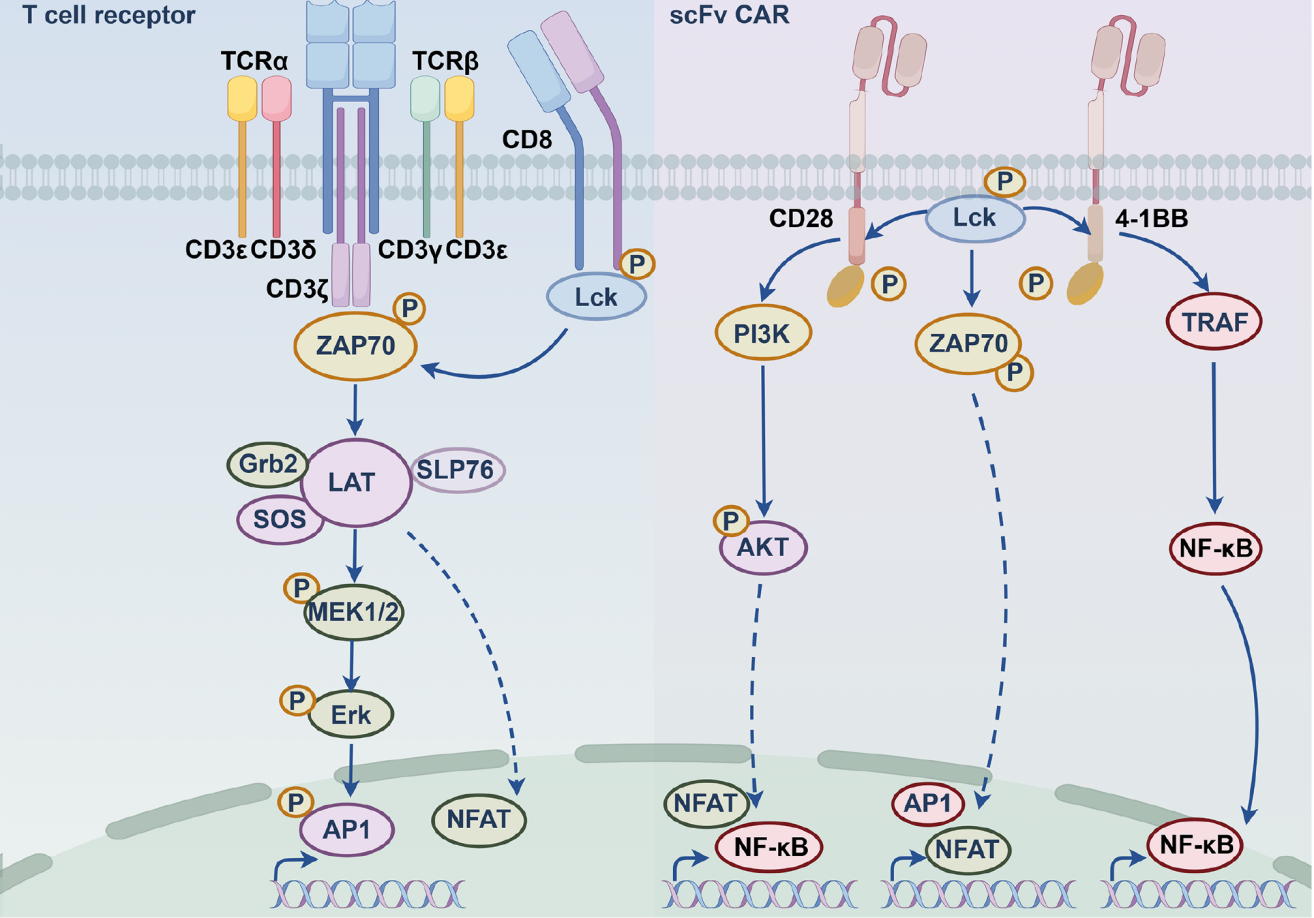
Figure 1 TCR versus CAR signaling: structural and functional divergence This figure contrasts the structures and downstream signaling cascades of the TCR and CAR. On the left, the TCR complex consists of α/β chains (for pMHC recognition) and CD3 subunits (with ITAMs). Upon pMHC binding, LCK phosphorylates CD3ζ ITAMs, recruits ZAP70, and activates pathways like Grb2-SOS-ERK and PI3K-AKT, with CD8 acting as a coreceptor. On the right, the modular CAR includes an extracellular antigen-binding domain (e. g., scFv), a hinge, a transmembrane domain, and intracellular modules (CD3ζ plus co-stimulatory domains like CD28 or 4-1BB). Antigen binding induces oligomerization; CD28 accelerates effector function, while 4-1BB activates NF-κB via TRAF to enhance T cell persistence. The figure also highlights key differences: TCR relies on MHC restriction, while CAR is MHC-independent.

In contrast to major histocompatibility complex (MHC)-restricted conventional T cells, CARs are typically designed to recognize non-MHC cell surface proteins [13]. Upon antigen binding, CARs undergo oligomerization, leading to the phosphorylation of the intracellular CD3ζ immunoreceptor tyrosine-based activation motif (ITAM) by lymphocyte cell-specific protein-tyrosine kinase (LCK). Zeta-chain-associated protein kinase 70 (ZAP-70) is subsequently recruited and phosphorylates many known substrates, including LAT, SLP-76, and PLC-γ, upon CAR ligation [ 14, 15] . Additionally, the CAR structure includes 1–2 co-stimulator domains. The most frequently used domains are derived from the CD28 family (like CD28) or the tumor necrosis factor receptor (TNFR) family (like 4-1BB). Most studies of CAR signaling have shown that CD28-containing CARs signal more rapidly and intensely than 4-1BB-containing CARs by facilitating CD3ζ phosphorylation by LCK [16]. 4-1BB-containing CARs recruit the THEMIS/SHP1 phosphatase complex to attenuate CD3ζ-chain phosphorylation [16]. In addition, they also induce different signaling pathways, which will be discussed in detail later ( Figure 1).

However, this integrated and simplified architecture may result in less precise signal modulation than endogenous TCRs do. For example, in a study comparing GD2-targeted CAR variants in varicella zoster virus-specific T cells, the GD2-CAR.CD28ζ-modified cells showed robust proliferation upon TCR restimulation, whereas those lacking co-stimulatory domains or with 4-1BB signaling had impaired expansion and endogenous TCR function [17]. Additionally, tonic signaling, which causes spontaneous receptor activation and accelerates T-cell exhaustion, is a significant challenge in CAR-T-cell therapy. Positively charged patches (PCPs) on the CAR’s antigen-binding domain drive this abnormal activation by inducing receptor clustering. Strategies such as reducing PCPs or increasing ionic strength during *ex vivo* expansion can suppress this signaling and reduce exhaustion [18].

### TCR signaling

The core TCR complex consists of two TCR chains and six CD3 chains (two ε chains, one γ chain, one δ chain and two ζ chains) [19]. Additionally, several other components, including coreceptors, kinases, and ligands, play essential roles in the TCR signaling pathway [20]. More than 95% of mature T cells express TCRα and TCRβ isoforms [21]. In this review, we focus on αβ T-cell signaling.

The TCR binds to its specific antigenic peptide present in the groove of the MHC molecule, and this binding is strengthened by mechanical forces that facilitate conformational changes and enhance affinity [22]. Recent studies have shown that agonist peptides form catch bonds with TCRs, where force increases the bond lifetime, thereby increasing calcium signaling and T cell activation [23]. This mechanotransduction is essential for precise antigen discrimination and initiating immune responses [24]. Structurally, TCRs function as anisotropic mechanosensors: the applied force generates torque on CD3 subunits, triggering intracellular signaling through lipid bilayer deformation. This process depends on the TCR structure, lipid environment, and other biochemical factors [25]. TCR activation involves phase separation between CD3ε and LCK via ionic interactions, forming condensates that increase LCK-mediated CD3 phosphorylation in ITAMs and amplify signaling. The binding of the LCK SH3 domain to the exposed RK motif resulted in local augmentation of LCK activity and CD3 phosphorylation [26]. Interestingly, this process is tightly regulated, as phosphorylated CD3ε recruits Csk to dissolve the condensate and inhibits LCK [ 27– 29] . ZAP70 kinase is subsequently recruited and activated through transphosphorylation, leading to LAT phosphorylation and the assembly of the LAT-SLP76 signalosome, which activates downstream pathways [ 30– 32] . Additionally, the TCR signaling cascade is regulated, with phosphorylated LAT forming liquid-phase condensates enriched in PLCγ1 [33], SOS1 [34], GRB2 [35], and GADS [36], driving ERK/MAPK activation [37] and SLP76 phosphorylation within minutes of stimulation [38]. In combination with co-stimulation signaling, such as CD28-B7, T cell proliferation capacity and effective function, including cytotoxicity, are increased ( Figure 1).

Mechanical forces actively modulate T cell activation through TCR-pMHC interactions, influencing the specificity and magnitude of the immune response. They facilitate the dynamic translocation of TCR and MHC, which is essential for the initiation and propagation of TCR signaling. This interplay enhances TCR-MHC interaction stability and modulates the subsequent intracellular signaling cascade. For instance, studies have demonstrated that TCRs can be activated by monovalent ligands only when these ligands are membrane associated, underscoring the importance of mechanical forces in TCR activation [39]. Recent studies have indicated that β2 integrin mechanical signaling can separate T cell proliferation from differentiation, resulting in the production of stem cell-like CAR-T cells. β2 integrin activation recruits 14-3-3ζ, causing YAP phosphorylation and inactivation. This inactivation of YAP releases the transcription factor MafG, which activates stemness genes, thus generating therapeutic stem cell-like CAR-T cells [40]. Additionally, T cells can sense the mechanical rigidity of their microenvironment, with stiffer matrices enhancing T cell activation and proliferation [41]. The TCR acts as a mechanoreceptor, with local membrane bending increasing TCR signaling domain accessibility to phosphorylation, facilitating selective agonist recognition [42]. DNA origami tension sensors reveal that TCR-antigen bonds experience forces in the range of 5--10 pN, which are influenced by factors such as the cell state and F-actin activity [43]. By understanding these mechanical aspects, we can gain deeper insights into how T cells recognize and respond to antigens, which is fundamental to immune response initiation.

TCR-T-cell therapy involves engineering T cells to express a specific TCR that can recognize a tumor-associated antigen presented on MHC molecules. TCR-T therapy has shown remarkable efficacy in tumor models. For example, TBI-1301 achieved a 50% objective response rate in synovial sarcoma [44], and TCR-T cells targeting melanoma antigens such as MART-1 have demonstrated significant tumor regression [45]. However, the utility of TCR-T cells remains dependent on MHC-I expression and the tumor antigens they present. Some epithelial tumors often downregulate MHC-I expression, rendering TCR-T cells ineffective at recognizing and targeting these cells [46]. Additionally, identifying tumor-specific TCR remains challenging [47].

## Co-stimulatory Domains in CAR-T Cells: CD28, 4-1BB, CD27, ICOS, OX40, and Others

While CAR-T immunotherapy has shown excellent potential for treating hematological malignancies, breakthroughs in the field of solid tumors are urgently needed [48]. A critical component of CAR design is the inclusion of co-stimulatory domains, which enhance T-cell activation, proliferation, and persistence. For example, incorporating specific signaling domains, such as CD28 and 4-1BB, can significantly increase CAR-T cell persistence and functionality [49]. Additionally, many different co-stimulatory domains lead to distinct signaling mechanisms and varying clinical efficacy.

### CD28: potent effector responses to accelerated exhaustion

The CD28 co-stimulatory domain, which is widely used for its strong activation signals, greatly impacts downstream signaling pathways in CAR constructs, enhancing CAR-T-cell anti-tumor efficacy. It functions primarily by enhancing CD3ζ phosphorylation kinetics, leading to increased ERK activation via the MAPK pathway, which is essential for T cell activation and function [50]. CD28 co-stimulation also augments CAR signaling via the PI3K-AKT [51] pathway and promotes T-cell glycolysis [52]. CD28 co-stimulation also enhances CAR signaling through the LCK/CD3ζ/ZAP70 axis, increasing their anti-tumor efficacy and long-term cytotoxicity [53].

The incorporation of CD28 signaling into CAR designs has been shown to significantly increase T-cell anti-tumor activity. CD28 co-stimulation has been shown to enhance trafficking, expansion, and tumor control in solid tumors such as neuroblastoma and ovarian carcinoma [54]. It was also found to be more effective than 4-1BB in promoting CAR-T-cell expansion at the tumor site and inducing tumor regression in preclinical models that target antigens such as L1CAM and HER2 [54]. Its ability to inhibit the CD73-mediated regulatory activity of CD8 ^+^ T cells is crucial, as it prevents these cells from becoming suppressive in the tumor microenvironment and thus strengthens the overall immune response against tumors [55]. In addition to its role in CAR-T-cell therapy, CD28 plays a significant role in other immunotherapeutic approaches. CD28 co-stimulation is superior to 4-1BB co-stimulation in generating CAR-natural killer (CAR-NK) cells, resulting in better activation, cytotoxicity, and *in vivo* anti-tumor efficacy [56]. The development of bispecific antibodies such as NI-3201, which mediate PD-L1-dependent CD28 co-stimulation, provides a novel therapeutic option by enhancing T cell activity and anti-tumor function through dual mechanisms [57]. Moreover, CD28 plays a critical role in PD-1-targeted therapies, as the CD28-B7 co-stimulatory pathway is indispensable for rescuing exhausted CD8 ^+^ T cells during PD-1 blockade [58]. Thus, the integration of CD28 signaling into immunotherapeutic strategies is highly promising for enhancing therapeutic outcomes [ 59, 60] .

A primary drawback is that, compared with those with co-stimulatory domains such as 4-1BB, CD28-based CAR-T cells often exhibit limited *in vivo* sustainability [ 61– 64] . The rapid clearance of CD28 CAR-T cells is often attributed to T-cell exhaustion, where T cells lose their functional capabilities over time. Additionally, it is associated with increased regulatory T cell (Treg) infiltration, which can suppress antitumor immunity. Studies have shown the reduced efficacy of CD28-based CAR-T cells in solid tumors because Treg cells hinder T-cell-mediated attacks on cancer cells [65]. CD28 co-stimulation also increases the risk of cytokine release syndrome (CRS) [ 66, 67] . Researchers have identified specific amino acid residues within the CD28 domain that contribute to this exhaustion, and modifying these residues has been shown to increase the durability of the anti-tumor response [ 68, 69] . These findings suggest that while CD28 induces strong effector responses, strategies are needed to mitigate its associated accelerated exhaustion and overcome the inhibitory effects of Treg cells in solid tumor immunotherapy.

### 4-1BB: metabolic resilience and long-term persistence

The 4-1BB co-stimulatory domain, a member of the TNFR superfamily and known as CD137, plays a pivotal role in enhancing CAR-T-cell efficacy and persistence [ 70, 71] . When CAR binds to an antigen, 4-1BB binds to the TNF receptor-associated factor (TRAF) family to modulate the activation of the canonical and noncanonical nuclear factor κB (NF-κB) pathways [ 51, 72] . The NF-κB pathway protects T cells from apoptosis by inducing anti-apoptotic genes ( *e*. *g*., Bcl-2 and cIAPs) [ 73, 74] . It also modulates the Fas-mediated apoptosis pathway, sometimes increasing tumor cell sensitivity to apoptosis [ 75, 76] . Moreover, the interaction between 4-1BB and TRAF not only enhances NF-κB signaling but also contributes to the formation of protein complexes that are distinct from those formed during TCR signaling [77]. It provides signals that increase T-cell survival, proliferation, and anti-tumor activity [ 70, 78, 79] .

The inclusion of the 4-1BB co-stimulatory domain in CAR-T cells has been shown to increase their persistence and anti-tumor efficacy. Studies have demonstrated that, compared with CAR-T cells with CD28 co-stimulation, 4-1BB-based CAR-T cells exhibit a less differentiated memory status, which is associated with improved perseverance and proliferation [80]. In addition, research comparing CD19 CAR-T cells with CD28 co-stimulated and 4-1BB co-stimulated (BBζ) domains revealed that BBζ CAR-T cells exhibited greater *ex vivo* survival and expansion [72]. This was attributed to the activation of noncanonical nuclear factor κB (ncNF-κB) signaling in BBζ CAR-T cells [81]. This domain also reduces T-cell exhaustion, a common issue in CAR-T cell therapy for solid tumors, by ameliorating tonic signaling, thereby improving overall anti-tumor efficacy [ 62, 81– 83] . Furthermore, the 4-1BB co-stimulatory domain positively influences the metabolic state of T cells. Compared with predominant oxidative metabolism, CD28-mediated costimulation results in pronounced glycolytic metabolism and increased susceptibility to exhaustion and lower susceptibility to exhaustion of 4-1BB-mediated co-stimulation [52]. Additionally, compared with CD28-based CARs, 4-1BB co-stimulated CAR-T cells enhance immune synapse formation and proinflammatory gene activation more effectively. This is due to its autonomous signaling and receptor homodimerization capability, which boosts anti-tumor responses and persistence [84].

In solid tumors, the tumor microenvironment (TME) presents challenges such as immunosuppressive factors and physical barriers, limiting T-cell infiltration and function. While 4-1BB signaling in CAR-T cells supports T-cell survival and proliferation, it may not overcome the hostile TME, leading to suboptimal anti-tumor responses. Compared with CD28 co-stimulation, which offers stronger initial T-cell activation, 4-1BB-based CAR-T cells often show weaker antitumor activity in solid tumors, where a more aggressive immune response is needed [80]. Additionally, the persistence of 4-1BB co-stimulated CAR-T cells can result in prolonged exposure to the immunosuppressive TME, causing T-cell exhaustion, characterized by reduced cytokine production and cytotoxic activity, which further diminishes therapeutic potential [62]. In addition, 4-1BB-containing CARs recruit the THEMIS/SHP1 phosphatase complex to attenuate CD3ζ-chain phosphorylation [16], which slows CAR-T-cell function, leading to BBζ CAR-T cells having limited efficacy against low-antigen-expressing targets compared with CD28 co-stimulated CAR-T cells [85]. 4-1BB in CARs causes cell aggregation and death without CAR tonic signaling. Studies have shown that 4-1BB sequesters A20 at the cell membrane in a TRAF-dependent manner, resulting in A20 deficiency. This leads to NF-κB hyperactivity, ICAM-1-driven cell aggregation, and cell death via RIPK1/RIPK3/MLKL. Genetic modifications such as overexpressing A20 or deleting the TRAF-binding motif of 4-1BB can prevent cell clustering and death, enhancing the anti-tumor ability of 4-1BB co-stimulated CAR-T cells [51].

### CD27: bridging innate and adaptive immunity

CD27, a member of the tumor necrosis factor receptor superfamily, plays a key role in T cell activation by providing a co-stimulatory signal crucial for effective immune responses [86]. Its interaction with CD70 enhances T cell proliferation and differentiation into effector and memory T cells, making it a promising target in cancer immunotherapy [ 87, 88] . CD27 could recruit the signaling adaptor TRAF2 and the phosphatase SHP-1, thereby modulating TCR and CD28 signals. The modulation of TCR signals promotes transcription factor circuits that induce memory-associated rather than effector-associated gene programs [89]. In addition, CD27 signaling can also activate *de novo* nucleotide and protein synthesis [90] and Pim kinase [91] signaling pathways, leading to the regulation of T cell expansion, differentiation, and effector functions. Overall, the CD27 co-stimulatory domain significantly enhances the survival and anti-tumor activity of redirected human T cells [ 92, 93] .

CD27 as a co-stimulatory domain in CAR-T cells, enhances anti-tumor efficacy *in vivo*, improving human cancer regression in xenogeneic allograft models. [87]. This is due to increased antigen-stimulated effector functions ( *e*. *g*., cytokine secretion, cytotoxicity), proliferation, and resistance to apoptosis. *In vivo*, CD27-bearing CAR-T cells have increased persistence post-infusion, leading to better cancer regression [87]. Additionally, CD27-costimulated CAR-T cells achieve better tumor control than CD28-costimulated CAR-T cells do by promoting memory-relevant properties [89].

Further research has demonstrated that CD27 signaling can be effectively combined with other co-stimulatory signals to improve CAR-T-cell efficacy. For example, combining CD27 with CD28 and 4-1BB signaling in CAR-T cells targeting colorectal tumors resulted in enhanced proliferation, reduced expression of immune checkpoint receptors, and increased generation of memory stem T cells, thereby improving anti-tumor capacity in xenograft models [ 88, 92] . Another example is the use of a bispecific antibody targeting both CD27 and EGFR, which induces cancer cell-localized crosslinking and activation of CD27, thereby enhancing T-cell cytotoxicity and proliferation in EGFR-expressing cancers [94]. These approaches highlight the potential of CD27 co-stimulation in conjunction with targeted therapies to overcome the immunosuppressive barriers of solid tumors.

CD27 activates pathways vital for T-cell responses but faces challenges in the TME, especially in solid tumors. Its interaction with CD70 can increase the number of Tregs, thus suppressing tumor-specific T-cell responses. This may promote tumor growth by increasing angiogenesis and reducing Treg apoptosis, creating an immunosuppressive environment [95]. Thus, CD27 might enhance immune responses but also increase the number of Tregs and support tumor growth, posing a challenge for solid tumor therapies. This highlights the need for caution and strategies to counteract these effects when CD27 co-stimulatory domains are used in CAR-T cells.

### ICOS: tailoring helper T-cell responses

ICOS, a member of the TNF receptor superfamily, plays a crucial role in tailoring helper T-cell responses, particularly in CAR-T-cell therapies [96]. It is expressed on activated T cells and interacts with ICOS-L, which is found on antigen-presenting cells and tumor cells within the TME [ 97, 98] . This interaction induces the recruitment of PI3K, leading to the production of membrane-bound phosphatidylinositol 3,4,5-trisphosphate (PIP _3_), culminating in the activation of Akt to promote T cell proliferation and survival [ 99, 100] . This signaling pathway has a dual role, both in promoting anti-tumor immunity and potentially facilitating tumor progression through Treg activity [101]. Recent studies have highlighted the potential of ICOS as a biomarker for predicting response to immunotherapy [102]. ICOS expression on T cells is correlated with better outcomes in patients receiving immune checkpoint blockade therapies targeting PD-1 and CTLA-4 [ 103, 104] . The presence of ICOS ^+^ T cells in the TME indicates increased immune activity and better clinical responses, suggesting that ICOS could be an indicator of effective T cell-mediated immune responses in cancer patients [ 105– 107] . In CAR-T-cell therapy, integrating ICOS into constructs enhances cytotoxicity and promotes T-cell persistence, which is crucial for sustained anti-tumor activity [ 108, 109] .

When combined with other co-stimulatory domains, such as OX40, ICOS has been shown to enhance CAR-T-cell cytotoxicity and promote a T-cell persistence phenotype, improving anti-tumor activity in both *in vitro* and *in vivo* models. These findings suggest that ICOS can significantly contribute to the long-term efficacy of CAR-T cells in the solid TME [110]. The dual co-stimulation approach improves CAR-T cell anti-tumor efficacy and persistence, as shown in studies with dual anti-CD19/CD20 CAR constructs [111]. The unique signaling pathways of ICOS contribute to a more robust immune response, potentially addressing the limitations of some CAR-T cells in solid tumors [112]. The molecular mechanisms by which co-stimulatory domains such as ICOS enhance CAR-T-cell function are being elucidated, offering insights into optimizing these domains for better therapeutic outcomes [78]. Additionally, ICOS can influence the tumor microenvironment; Tregs expressing CARs with ICOS domains have shown potential in controlling allograft rejection [113], indicating that ICOS can modulate immune responses in complex tissue environments. This ability to integrate native and CAR-mediated co-stimulatory signals underscores the versatility of ICOS in enhancing CAR-T-cell therapies for solid tumors [114].

In addition to CAR-T cells, ICOS plays a role in the immune system, such as in anti-atherosclerotic processes, by downregulating vascular smooth muscle phagocytosis and proliferation [115]. These findings suggest that ICOS can modulate immune responses in a broader context, which could benefit comprehensive cancer immunotherapies. ICOS expression also serves as an indicator of T cell-mediated responses to cancer immunotherapy. A single ICOS co-stimulator can enhance T cell proliferation and survival but may not provide optimal co-stimulatory signals for effective anti-tumor activity in solid tumors. These tumors often have a hostile microenvironment with immunosuppressive factors, physical barriers, and a lack of suitable antigens. Although ICOS promotes T-cell activation, it may not adequately overcome these barriers, leading to suboptimal CAR-T-cell performance. Moreover, ICOS expression can be downregulated in certain TMEs, further limiting its effectiveness [115]. Additionally, the use of ICOS as a co-stimulatory domain in CAR-T cells may inadvertently promote regulatory Treg activity, which can suppress the immune response against tumors. This is particularly concerning in solid tumors, where Tregs are often linked to a poor prognosis. The potential for ICOS to enhance Treg function could counteract the therapeutic effects of CAR-T cells, making it a less favorable choice for solid tumor applications [116]. Further research is needed to optimize co-stimulatory domains for CAR-T-cell therapy in solid tumors, possibly by combining ICOS with other domains or modifying ICOS signaling pathways to better suit the complex landscape of solid malignancies.

### OX40

OX40, a TNFR superfamily member, plays a critical role in T cell activation, proliferation, and survival, promoting the expansion and effector differentiation of both CD4⁺ and CD8⁺ T cells [ 117, 118] . When included in CAR constructs, OX40 can increase T-cell proliferation and survival through pathways such as the NF-κB, MAPK, and PI3K-AKT [119]. Research has shown that OX40 co-stimulation enhances CAR-T cell persistence when CAR-T cells are repeatedly stimulated by target cells. This is critical for reducing relapse rates after CAR-T-cell therapy. Both *in vitro* and *in vivo* studies have shown that OX40 CAR-T cells have greater proliferation and enhanced immune memory than other co-stimulatory domains, such as 4-1BB, making them promising options for improving the durability of CAR-T-cell responses [ 120– 122] . Additionally, OX40 co-stimulation can reverse tumor-induced T-cell anergy, a state that limits anti-tumor immunity. By engaging OX40, CAR-T cells can restore the proliferative capacity of tumor-reactive T cells, reduce tumor growth, and improve the survival of tumor-bearing hosts [118]. Moreover, OX40 signaling can increase CD4 ^+^ and CD8 ^+^ T-cell clonal expansion and effector differentiation, which are crucial for effective tumor elimination [117]. Furthermore, OX40 co-stimulation can suppress the secretion of inhibitory cytokines such as IL-10, which otherwise weakens T-cell-mediated immunity while preserving the production of pro-inflammatory cytokines [123]. In multiple myeloma, a hematologic cancer, OX40 integration into CAR-T cells has yielded promising results. BCMA-BBZ-OX40 CAR-T-cell therapy, which was generated via a rapid manufacturing platform, demonstrated enhanced cytotoxicity and reduced exhaustion in preclinical studies. It also improved proliferation and T-cell stemness, leading to strong responses in patients with relapsed/refractory multiple myeloma, with a high overall response rate, and many patients achieved complete responses [124]. These findings highlight the potential of OX40 in overcoming the immunosuppressive tumor microenvironment in solid tumors.

### Emerging co-stimulators: CD2, GITR, and HVEM

#### CD2

CD2, expressed on T and NK cells, is a co-stimulatory protein crucial for cell adhesion and recognition. Its interaction with CD58 is essential for T cell activation and the formation of immunological synapses between T cells and antigen-presenting cells, playing a key role in effective immune responses against tumors. However, in some cancers, such as diffuse large B-cell lymphoma, downregulation of CD58 on tumor cells can reduce T-cell activation and function, and low CD58 expression is linked to shorter progression-free survival. Additionally, TILs with reduced CD2 expression are associated with defective cytotoxicity and T cell exhaustion, complicating the immune response in non-hematological neoplasms [125]. CD2 and CD28 co-stimulation trigger overlapping yet distinct signaling pathways. CD28 co-stimulation is particularly important for proper NF-κB activation, whereas CD2 signals more strongly to the S6-ribosomal protein [126].

CD2 co-stimulation enhances T cell activation, proliferation, and survival. CD2 can compensate for the absence of other co-stimulatory domains and enhance CAR-T-cell functional activity, offering advantages in solid tumors where strong T-cell activation is needed to overcome the immunosuppressive TME [127]. However, the use of CD2 as a co-stimulatory domain in CAR-T cells presents challenges. One issue is the risk of on-target off-tumor effects due to CD2 ligand expression on normal tissues [128]. Additionally, the efficacy of CD2-mediated co-stimulation in solid tumors is still under investigation, and more clinical data are needed to fully understand its benefits and limitations. Current research focuses on balancing effective tumor targeting with minimizing potential toxicity. Preclinical studies have shown that CAR-T cells incorporating CD2 co-stimulatory domains can produce high levels of IL-2 and exhibit increased proliferation and persistence [127]. In parallel, the development of an allogeneic CD2-deleted UCART2 for T-cell malignancies revealed that CD2 deletion affects CAR-T-cell function. However, this effect can be compensated for by rhIL-7-hyFc, thereby improving efficacy and survival [129].

#### GITR

Glucocorticoid-induced TNFR-related protein (GITR) is a co-stimulatory receptor that plays a significant role in modulating immune responses and cancer immunotherapy. Its activation enhances effector T cell function while inhibiting Tregs, promoting anti-tumor immunity. This dual role makes GITR a promising target for solid tumor immunotherapy. GITR activates T cells and inhibits Treg-mediated suppression. When activated by its ligand or agonistic antibodies, GITR increases CD8 ^+^ and CD4 ^+^ T cell proliferation and activation and depletes or impairs Tregs in the tumor microenvironment, further enhancing tumor targeting [ 130, 131] .

Studies have shown that incorporating GITR into CAR-T cells can increase anti-tumor efficacy by promoting T-cell proliferation and survival and reducing T-cell exhaustion, which is common in the tumor microenvironment [81]. The unique signaling pathways of GITRs can lead to a more robust and sustained immune response against tumors. However, the use of GITR in CAR-T cells has potential disadvantages. There is a risk of on-target off-tumor cytotoxicity, where CAR-T cells might attack normal tissues with low-level target antigen expression. Additionally, the complex interplay between GITR signaling and other immune pathways can lead to unpredictable outcomes, requiring careful CAR construct design and testing [ 113, 132] . Preclinical studies have shown promising results, with GITR-enhanced CAR-T cells demonstrating improved persistence and anti-tumor activity in various solid tumor models [133]. However, translating these findings into clinical success needs further research to balance efficacy and safety.

#### HVEM

The herpes virus entry mediator (HVEM) is a significant player in the immune response and has been identified as a potential target in cancer immunotherapy, particularly in solid tumors. As a co-stimulatory molecule that mediates both stimulatory and inhibitory signals, HVEM is an attractive therapeutic target. Its role in tumor biology, particularly in NSCLC, reveals that HVEM can suppress metastasis by inhibiting glycolysis and modulating macrophage polarization [134]. Interaction with B and T lymphocyte attenuators (BTLAs) could enhance anti-tumor immunity. BTLA is involved in immune escape mechanisms, where upregulated BTLA can weaken T cell responses against cancer. Targeting this axis could reverse tumor-induced T cell dysfunction, providing a new avenue for cancer immunotherapy [135].

In CAR-T-cell therapy, high BTLA expression is correlated with poor clinical response. However, deleting BTLA in CAR-T cells has improved tumor control, indicating that the BTLA/HVEM axis is a crucial immune checkpoint in CAR-T-cell immunotherapy [136]. HVEM co-stimulation enhances CAR-T-cell function through a novel downstream signaling pathway that increases metabolic activity. This metabolic reprogramming is crucial, as it endows CAR-T cells with improved function and persistence within the hostile TME. Studies have shown that HVEM CAR-T cells exhibit more robust cytokine release and cytotoxicity than traditional co-stimulatory domains (CSDs), such as 4-1BB or CD28 [112]. This enhanced functionality is attributed to the ability of HVEM to attenuate T-cell exhaustion, a common challenge in the treatment of solid tumors [137]. HVEM CAR-T cells maintain low levels of exhaustion even after prolonged antigen-dependent proliferation and efficiently differentiate into central and effector memory subsets, which is associated with elevated energy metabolism and reduced exhaustion [138]. Despite these advantages, the use of HVEM in CAR-T cells has potential disadvantages and challenges. The novel signaling pathways activated by HVEM may cause unforeseen off-target effects or toxicities, which requires thorough preclinical and clinical investigations. Additionally, the long-term persistence and safety of HVEM CAR-T cells in patients need evaluation to ensure that no adverse effects or autoimmune responses occur.

### Common logic versus distinctive traits

All co-stimulatory domains enhance T cell activation and anti-tumor efficacy by increasing CD3ζ ITAM phosphorylation and converging on PI3K or TRAF/NF-κB signaling, influencing therapeutic outcomes through signal strength, duration, and metabolic coupling. CD28 offers high-intensity/short-lived signals for potent cytotoxicity but risks rapid exhaustion, whereas 4-1BB provides modest-intensity/long-lived signals, supporting metabolic resilience and memory formation. Intermediate modules such as CD27 and OX40 balance these traits. ICOS selectively activates Akt through PIP3 production, fine-tuning helper T cell responses. GITR activates the NF-κB and MAPK pathways with varying intensities and durations. HVEM uniquely balances stimulatory and inhibitory signals, offering potential for metabolic reprogramming in the TME. CD28-based CAR-T cells show superior initial anti-tumor activity in hematological cancers but limited persistence in solid tumors due to rapid exhaustion. 4-1BB CAR-T cells demonstrate long-term persistence but may require antigen density thresholds for effective activation. CD27 and ICOS CAR-T cells show promise in preclinical models but require careful ligand expression analysis to avoid unintended immune modulation. OX40 and GITR CAR-T cells offer novel mechanisms for overcoming T-cell anergy and exhaustion but require validation in diverse solid tumor contexts. HVEM CAR-T cells present unique metabolic advantages but need thorough evaluation of off-target effects and long-term safety profiles.

The selection of co-stimulatory domains in CAR-T-cell design for solid tumor immunotherapy should balance the activation intensity, metabolic support, and counteraction of immunosuppressive mechanisms. CD28 or CD2 is suitable for the rapid reduction of large tumor burdens. For long-term tumor surveillance, domains supporting T-cell persistence, such as 4-1BB and HVEM, are preferred. In tumors with high Treg infiltration, combining GITR with CD28 enhances CAR-T-cell efficacy. For glucose-deprived TMEs, dual 4-1BB plus HVEM signaling provides metabolic advantages. These evidence-based design principles can guide the development of more effective, context-specific CAR-T-cell therapies for solid tumors. Please refer to Table 1 for a summary of the key features and limitations of each co-stimulatory domain.

**Table:** **Table 1** Side-by-side functional comparison of different co-stimulatory domains

Domain	Key adaptor/pathway	Exhaustion level	Cytokine profile	Persistence	Main limitation
CD28	PI3K-Akt, LCK/ZAP70	Rapid	Th1 + Th2	Short	CRS risk; Treg recruitment
4-1BB	TRAF, NF-κB (ncNF-κB)	Pronounced	Th1	Long	Slow kinetics; poor low-antigen killing
CD27	TRAF2–SHP1 memory circuit	NA	Th1 + Th17	Medium to Long	CD70-mediated Treg expansion
ICOS	PI3K-Akt-Pim	NA	Th1 + Tfh	Long	May empower Treg
OX40	TRAF-NF-κB	NA	Th1 + Th9	Long	Systemic T-cell activation
CD2	CD58-S6 ribosomal	NA	Th1	NA	Broad normal-tissue ligand
GITR	TRAF-NF-κB	NA	Th1	Long	Treg depletion linked to systemic inflammation
HVEM	BTLA checkpoint release	Low	Th1 + CTL	Long	Unknown off-target toxicities

## Current Clinical Trials of CAR-T Cell Therapies in Solid Tumors

Understanding CAR signaling is crucial for determining the outcomes of solid tumor immunotherapy. TCRs function through MHC-restricted, mechanosensitive activation, whereas CARs, with their synthetic design, bypass the limitations of antigen presentation. These distinct mechanisms influence T cell activation, durability, and tumor infiltration, which in turn influence the efficacy of immunotherapy. Different modifications of CAR signaling have been extensively studied in clinical trials focused on T-cell therapy for solid tumors. Here, we summarize the current clinical trials on CAR-T cells in solid tumors in Table 2 and provide an in-depth analysis of the key trends and insights derived from these trials.

**Table:** **Table 2** Current clinical trials of CAR-T cell therapies in solid tumors

Antigen	Tumor target	Sponsor	Phase	NCT number	Study start
IL13Ra2, EGFR	Glioblastoma	University of Pennsylvania	Phase 1	NCT05168423	2023-02-24
EGFR/B7-H3	Advanced solid tumors	Second Affiliated Hospital of Guangzhou Medical University	Early Phase 1	NCT05341492	2022-05-01
EGFR/TGFβR-KO	Advanced solid tumors	Chinese PLA General Hospital	Phase 1	NCT04976218	2022-03-15
EGFRvIII	Glioblastoma	Marcela V. Maus, MD, PhD	Phase 1	NCT05660369	2023-03-22
EGFRvIII	Glioblastoma	Hideho Okada, MD, PhD	Phase 1	NA	2024-04-30
CEA	Colorectal carcinoma	Chongqing Precision Biotech Co., Ltd	Phase 1 Phase 2	NCT04348643	2020-02-20
CEA	Colorectal carcinoma	Ruijin Hospital	Early Phase 1	NCT04513431	2020-08-30
CEA	Advanced solid tumors	Weijia Fang, MD	Phase 1	NCT05396300	2022-05-25
CEA	Advanced malignant tumors	Chongqing Precision Biotech Co., Ltd	Phase 1	NCT05415475	2021-09-10
CEA	Advanced malignant tumors	Chongqing Precision Biotech Co., Ltd	Phase 1 Phase 2	NCT05538195	2022-06-07
GPC3	Hepatocellular carcinoma	Zhejiang University	Phase 1	NCT05155189	2021-12-09
GPC3	Hepatocellular carcinoma	Zhejiang University	Phase 1	NCT04756648	2021-03-10
GPC3/MSLN	Advanced malignant tumors	Second Affiliated Hospital of Guangzhou Medical University	Phase 1	NCT06196294	2024-05-10
ROR1	NSCLC, TNBC, ovarian carcinoma	Lyell Immunopharma, Inc.	Phase 1	NCT05274451	2022-03-29
GD2	Diffuse intrinsic pontine gliomas (DIPG), spinal diffuse midline glioma (DMG)	Crystal Mackall, MD, Stanford University	Phase 1	NCT04196413	2020-06-04
GD2	Diffuse midline glioma (DMG), refractory CNS tumors	Baylor College of Medicine	Phase 1	NCT04099797	2020-02-03
GD2/PSMA	Recurrent or refractory solid tumors	Shenzhen Geno-Immune Medical Institute	Phase 1 Phase 2	NCT05437315	2022-06-30
GD2/CD70	Advanced malignant tumors	Shenzhen Geno-Immune Medical Institute	Phase 1 Phase 2	NCT05438368	2022-06-30
GD2/CD56	Advanced malignant tumors	Shenzhen Geno-Immune Medical Institute	Phase 1 Phase 2	NCT05437328	2022-06-30
CLDN18.2	Gastric/esophagogastric conjugate adenocarcinoma, pancreatic cancer	CARsgen Therapeutics Co., Ltd	Phase 1 Phase 2	NCT04581473	2020-10-23
CLDN18.2	Gastric cancer, gastroesophageal junction cancer, pancreatic cancer	Peking University	Phase 1	NCT03874897	2019-03-26
CLDN18.2	Gastric/gastroesophageal junction (GC/GEJ), pancreatic cancer (PC)	CARsgen Therapeutics Co., Ltd	Phase 1 Phase 2	NCT04404595	2020-10-23
CLDN18.2	Gastric cancer (GC)	Suzhou Immunofoco Biotechnology Co., Ltd	Phase 1	NCT05472857	2022-08-08
CLDN18.2	Advanced solid tumors	Shanghai East Hospital	Phase 1	NCT04467853	2020-09-21
CLDN18.2	Advanced solid tumors	Innovent Biologics (Suzhou) Co., Ltd	Phase 1	NCT05199519	2021-12-13
CLDN18.2	Pancreatic cancer	CARsgen Therapeutics Co., Ltd	Phase 1	NCT05911217	2023-07-11
CLDN18.2	Advanced solid tumors	Shenzhen University General Hospital	Not Applicable	NCT05620732	2022-10-01
CLDN18.2/PD-L1	Advanced solid tumors	Sichuan University	Phase 1	NCT06084286	2024-01-01
CLDN6	Recurrent or refractory solid tumors	BioNTech Cell & Gene Therapies GmbH	Phase 1	NCT04503278	2020-09-16
PSMA	Prostate cancer, salivary gland cancers	Poseida Therapeutics, Inc.	Phase 1	NCT04249947	2020-02-28
PSMA	Prostate cancer	Bioray Laboratories	Phase 1	NCT04053062	2020-07-16
PSMA/CD70	Advanced solid tumors	Shenzhen Geno-Immune Medical Institute	Phase 1 Phase 2	NCT05437341	2022-06-30
MSLN	Advanced refractory solid tumors	Shanghai Pudong Hospital	Phase 1	NCT05531708	2021-04-02
MSLN	Solid tumors	Shanghai Mengchao Cancer Hospital	Early Phase 1	NCT05373147	2020-10-30
MSLN	Solid tumors	Wuhan Union Hospital	Early Phase 1	NCT04489862	2020-05-13
MSLN	Recurrent or refractory solid tumors	Tongji Hospital	Early Phase 1	NCT05141253	2022-01-12
MSLN	Relapsed or refractory solid tumors	The First Affiliated Hospital with Nanjing Medical University	Early Phase 1	NCT05166070	2022-01-01
MSLN	Solid tumors	Shanghai Cell Therapy Group Co., Ltd	Early Phase 1	NCT04503980	2020-03-26
B7-H3	Advanced solid tumors	Shanghai Hansoh Biomedical Co., Ltd	Phase 1	NCT05276609	2021-11-28
B7-H3	Glioblastoma multiforme	Beijing Tiantan Hospital	Phase 1	NCT05241392	2022-01-27
B7-H3	Pediatric solid tumor	Seattle Children’s Hospital	Phase 1	NCT04483778	2020-07-13
B7-H3	Solid tumors	St. Jude Children’s Research Hospital	Phase 1	NCT04897321	2022-07-06
B7-H3	Advanced solid tumor	PersonGen BioTherapeutics (Suzhou) Co., Ltd	Phase 1	NCT05190185	2021-06-01
HER2	Advanced solid tumors	Bellicum Pharmaceuticals	Phase 1	NCT04650451	2020-12-07
HER2	Solid tumors	Shanghai PerHum Therapeutics Co., Ltd	Phase 1	NCT04511871	2020-07-09
HER2	Solid tumors	Baylor College of Medicine	Phase 1	NCT03740256	2020-12-14
HER2	Solid tumors	Sichuan University	Early Phase 1	NCT04684459	2021-03-12
HER2/TRAIL-R2	Metastatic breast cancer	Baylor College of Medicine	Phase 1	NCT06251544	2025-06
CD70	Relapsed or refractory renal cell carcinoma	CRISPR Therapeutics AG	Phase 1	NCT04438083	2020-06-16
CD70	Advanced/metastatic solid tumors	Chongqing Precision Biotech Co., Ltd	Phase 1	NCT05420545	2021-12-31
NKG2D/DR5	Solid tumors	Shenzhen Hospital, University of Chinese Academy of Sciences	Phase 1	MR-44-23-010294	2023-06-01
NKG2DL	Solid tumors	The Affiliated Hospital of the Chinese Academy of Military Medical Sciences	Early Phase 1	NCT05382377	2022-05-17
NKG2DL/CLDN18.2	Solid tumors	The Affiliated Hospital of the Chinese Academy of Military Medical Sciences	Phase 1	NCT05583201	2022-10-13
NKG2DL/CLDN18.2	Solid tumors	Peking University	Phase 1	NCT06134960	2023-11-16
OX40	Advanced solid tumors	Second Affiliated Hospital of Guangzhou Medical University	Phase 1	NCT04952272	2021-03-01
VEGFR1/PD-L1	Serosal cavity metastases of malignant tumor	Sichuan University	Phase 1	NCT05477927	2023-11-28
CD44/CD133/IL7Ra	Recurrent glioblastoma	Beijing Tiantan Hospital	Phase 1	NCT05577091	2023-09-30
GUCY2C	Advanced digestive system neoplasms	Beijing Immunochina Medical Science & Technology Co., Ltd	Early Phase 1	NCT05287165	2022-03-10

The clinical trials detailed in Table 2 highlight several key trends in the development of CAR-T-cell therapies for solid tumors. First, there is a growing emphasis on dual-antigen targeting strategies, such as those targeting GD2/PSMA and GD2/CD70, as seen in trials. These approaches aim to address the issue of antigen escape and enhance therapeutic efficacy by simultaneously targeting multiple tumor-associated antigens. Second, the integration of additional CARs that secrete cytokines or other effector molecules is becoming more prevalent. For example, trials incorporating HER2/TRAIL-R2 tandem CARs aimed to induce bystander cytotoxicity in antigen-negative tumor cells. Third, the use of gene editing, such as the disruption of endogenous checkpoints such as PD-1, to enhance CAR-T-cell function has been explored in several trials. This strategy aims to improve the persistence and anti-tumor activity of CAR-T cells in the immunosuppressive TME.

Moreover, the trials reflect the diversity of antigen targets being investigated, ranging from well-established targets such as EGFR and CEA to emerging targets such as CLDN18.2, B7H3, and CD70. The geographical distribution of these trials also indicates a global collaborative effort, with institutions in the United States, China, and Europe actively contributing to the advancement of CAR-T-cell therapy for solid tumors. Overall, these clinical trials represent critical steps toward optimizing CAR-T-cell therapies and overcoming the challenges associated with solid tumor immunotherapy. By learning from the signaling dynamics of TCRs and refining CAR designs through iterative clinical testing, researchers are paving the way for more effective and durable treatments for patients with solid malignancies.

## Challenges and Strategies of CAR-T Cell Therapies in Solid Tumors

**Figure 2:**
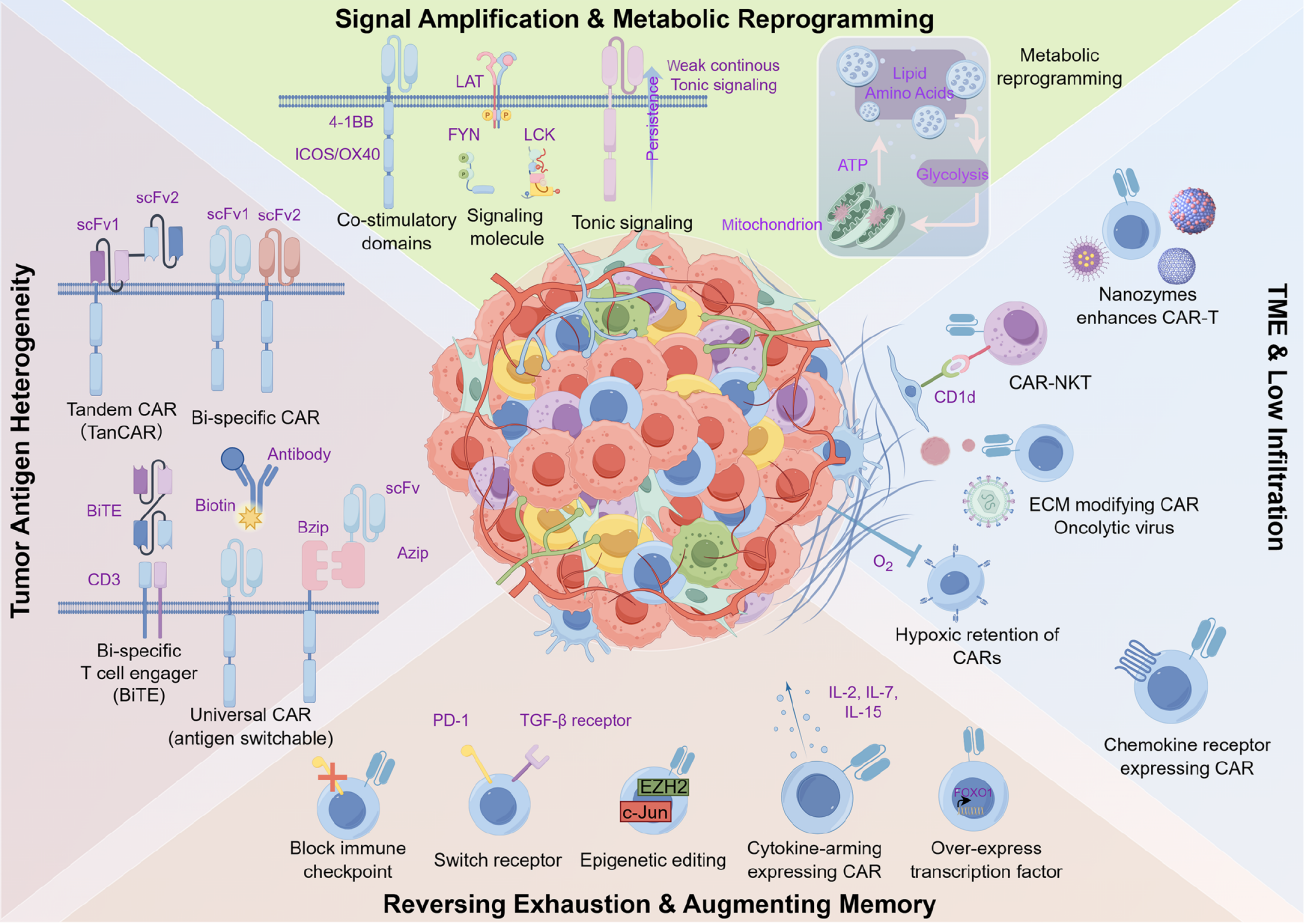
Figure 2 Challenges of CAR-T-cell therapies in solid tumors and strategies to overcome these challenges This figure outlines the main challenges of CAR-T cell therapy in solid tumors and corresponding solutions. Core challenges include poor CAR-T infiltration (due to tumor physical barriers), immunosuppressive TME (suppressive cells/cytokines, checkpoints), metabolic competition (tumor nutrient sequestration), tumor antigen heterogeneity/loss, and CAR-T exhaustion. Strategies to address these include engineering CAR-T cells to express chemokine receptors (for infiltration), armoring CAR-T cells to secrete pro-inflammatory cytokines (to reverse TME suppression), metabolic reprogramming (to enhance nutrient utilization), developing dual-target CARs (to counter antigen issues), and combining with checkpoint inhibitors (to reduce exhaustion).

While these trials are still ongoing, several challenges in solid tumors remain that hinder their clinical translation ( Figure 2).

### Low infiltration and suppressive microenvironments

After being introduced into cancer patients, CAR-T cells encounter numerous challenges imposed by the TME. The evasion of anti-tumor immune responses can begin even before the transferred T cells engage tumor cells, partly through mechanisms such as the CD47 “do not eat me” signal, which is overexpressed on tumors [139]. Unlike disseminated or circulating tumors, solid tumors establish physical barriers that impede T cell infiltration through a complex network of molecular and cellular mechanisms. These include the production of chemokines and other chemical cues that selectively attract immunosuppressive cells [140]; cancer-associated fibroblasts (CAFs) foster a dense, fibrotic milieu by irregularly depositing extracellular matrix (ECM) components [141], thus hampering T cell movement, and abnormal blood vessels with reduced expression of adhesion molecules crucial for T cell infiltration [ 142, 143] . To increase CAR-T-cell efficacy, strategies such as targeting the tumor vasculature to improve trafficking and infiltration have been proposed [144]. Additionally, genetic modifications of cytokine signaling pathways have been shown to increase CAR-T-cell infiltration and functionality within the TME [ 145, 146] . Additionally, targeting the tumor stroma, which often serves as a barrier to immune cell infiltration, has been shown to enhance CAR-T-cell accessibility and efficacy, offering potential for improving outcomes in solid tumor treatment [ 147– 149] . Moreover, combining CAR-T cells with oncolytic viruses has been demonstrated to increase their anti-tumor activity [150]. Through trafficking into solid tumors, CAR-T cells still face an immunosuppressive TME characterized by the infiltration of multiple immune cells, including myeloid-derived suppressive cells [151], tumor-associated macrophages [152], tumor-associated neutrophils [153], and Tregs [ 154, 155] . These suppressive cells can suppress CAR-T-cell function in several ways, including by producing immunosuppressive cytokines, upregulating receptors that inhibit T-cell activation, and creating an environment that is metabolically unfavorable for T cells [ 156– 158] .

Several innovative strategies have been developed to overcome these barriers. For example, owing to their photothermal and nanocatalytic properties, nanozymes can remodel the TME, helping to overcome immunosuppressive barriers. Notably, studies using B7-H3-targeted CAR-T cells in non-small cell lung cancer models have demonstrated that nanozyme treatment improves CAR-T cell infiltration and activation, resulting in enhanced anti-tumor activity and the transformation of the immune-hostile environment to support greater therapeutic efficacy [ 159– 164] . Additionally, emerging studies from others and our group have demonstrated that CAR-T-cell therapy based on natural killer T (NKT) cells results in superior anti-tumor activity *in vivo* via CD1d-dependent immune responses in the TME. CAR-NKT cells eliminate CD1d-expressing M2-like macrophages and myeloid-derived suppressor cells (MDSCs). In addition, CAR-NKT cells promote epitope spreading and the activation of endogenous T cell responses against tumor-associated neoantigens [ 150, 165] .

In addition, the hypoxic feature of the tumor microenvironment due to rapid cell proliferation and poor vascularization significantly impacts cellular metabolism and function. In T cells, hypoxia triggers a metabolic shift from oxidative phosphorylation to glycolysis to maintain ATP production under low-oxygen conditions. However, this adaptation can lead to T-cell exhaustion, thereby reducing their anti-tumor efficacy [ 166, 167] . Targeting metabolic pathways, such as modulating HIFs or inhibiting autophagy, presents a promising strategy to increase T-cell fitness and anti-tumor activity [168]. Recent studies have explored the engineering of T cells with hypoxia-inducible elements, such as hypoxia-inducible chimeric antigen receptors (HiCARs). HiCAR-T cells, which are activated under hypoxic conditions, show selective tumor cell targeting and reduced off-target effects, improving both the safety and efficacy of CAR-T-cell therapies in solid tumors [169]. Moreover, strategies to improve CAR-T-cell metabolism via the use of drugs that activate AMPK and inhibit mTOR, such as metformin and rapamycin, have also been shown to help overcome hypoxic conditions [170].

### Signal amplification to avoid dysfunction

The signal transduction domains (STDs) significantly impact CAR signal input efficiency. The incorporation of specific STDs from molecules such as CD3ζ and CD28 enhances cytokine secretion and cytotoxicity in CAR-T cells, highlighting the importance of the STD structure and signaling in modulating CAR activity [171]. However, inefficient CAR proximal signaling remains a bottleneck, reducing antigen sensitivity. Studies indicate that the inefficient recruitment and activation of key signaling proteins, such as ZAP-70, are significantly reduced in current CAR designs, limiting the efficacy of CAR-T-cell therapies, especially those that target tumors with low antigen expression [172].

To address these challenges, strategies such as integrating binding affinity and inputting signaling molecules have been proposed for rational CAR design. By adjusting the affinity of CARs and utilizing tonic signaling, researchers can increase T-cell functionality and sustain long-term tumor control, underscoring the need to balance CAR binding affinity and tonic signaling to optimize T-cell responses [173]. Another promising approach involves adjusting the charge density of CARs to optimize tonic signaling and CAR-T-cell fitness. Modifying the positively charged patches on the CAR antigen-binding domain can regulate CAR clustering and tonic signaling, potentially improving the *in vivo* persistence and anti-tumor function of CAR-T cells [18].

Moreover, compensating CAR-T cells with signaling molecules such as LCK, LAT, or FYN was demonstrated to efficiently overcome these barriers. LCK enhances T-cell activation and anti-tumor responses by phosphorylating signaling proteins and helps manage CAR-T-cell exhaustion and cytokine release [16]. LAT enhances CAR-T-cell sensitivity and persistence, particularly in antigen-low leukemias, through the ALA-CAR-T-cell platform, which enhances signaling and cytotoxicity [174]. FYN influences T-cell responses, and its modulation can promote regulatory T-cell differentiation, potentially reducing CAR-T-cell exhaustion and improving therapeutic outcomes [175].

In addition, the combination of two different co-stimulatory domains has been explored to enhance CAR-T cell function by balancing initial activation and long-term persistence. For example, the combination of CD28 with other co-stimulatory domains, such as 4-1BB, to enhance CAR-T-cell function by balancing initial activation and long-term persistence has been explored [ 176, 177] . The combination of 4-1BB with ICOS can improve the persistence and anti-tumor efficacy of CAR-T cells, providing a synergistic effect that enhances their therapeutic potential in solid tumors [108]. In solid tumors, combining OX40 with ICOS has been studied to further improve CAR-T-cell efficacy. This combination can promote a T-cell persistence phenotype and increase cytotoxicity against tumor cells, thereby increasing overall survival and long-term CAR-T-cell persistence *in vivo* [110]. These strategies show that combining two other co-stimulatory signals has the potential to overcome the limitations of CAR-T cell therapy in solid tumors [ 78, 81] .

### Metabolic reprogramming for nutrient competition

The clinical implications of TCR versus CAR signaling in solid tumors are profoundly influenced by metabolic reprogramming, which is a critical factor determining the efficacy of T cell-based therapies. In solid tumors, the metabolic landscape is often hostile to immune cells and is characterized by nutrient deprivation and the production of immunosuppressive metabolites. This hostile environment necessitates robust metabolic adaptation by T cells to maintain their anti-tumor activity [ 178, 179] . Recent studies have shown that targeting metabolic pathways such as the AMPK and PPAR pathways can overcome the restrictions of the tumor microenvironment [180]. Some transcription factors, such as Foxp3 and Foxo1, when introduced into advanced CAR-T cells, have been shown to reprogram cellular metabolism, reduce exhaustion, and enhance anti-tumor efficacy [ 181, 182] . These findings underscore the importance of metabolic reprogramming in overcoming the metabolic deficiencies induced by the TME, thereby enhancing the therapeutic potential of TCR/CAR-based therapies.

#### Mitochondrial remodeling

Mitochondrial remodeling is essential for effective immune responses. The dynamic processes of mitochondrial fusion and fission, supported by proteins such as Opa1, maintain the metabolic efficiency of memory T cells by promoting oxidative phosphorylation and fatty acid oxidation [183]. Moreover, overexpressing PGC-1α, a key regulator of mitochondrial biogenesis, enhances CD8 ^+^ T cell memory and anti-tumor immunity by increasing mitochondrial activity and metabolic fitness [ 184, 185] . These findings highlight the importance of mitochondrial dynamics in establishing and maintaining immunological memory, which is vital for effective vaccine responses and protective immunity against infections [ 186– 189] .

#### Restricted glycolysis

Inhibiting glycolysis in T cells also offers a promising strategy to increase their fitness and function in cancer immunotherapy. Glycolysis is essential for T cell activation and effector functions but can cause metabolic exhaustion. Targeting this pathway helps reprogram T cell metabolism, providing an edge in the nutrient-deprived tumor microenvironment where cancer cells often outcompete immune cells [ 190– 192] . Research has shown that inhibiting glycolysis can increase memory CD8 ^+^ T cell formation, which is crucial for sustained anti-tumor immunity. For example, the use of 2-deoxyglucose has been shown to promote memory T cell generation, thereby enhancing the immunotherapeutic potential of T cells against cancer [ 193, 194] . Furthermore, targeting tumor cell glycolysis can enhance checkpoint therapy efficacy, as observed in highly glycolytic tumors, where genetic glycolysis suppression improves outcomes [ 191, 192] . Overall, inhibiting glycolysis and reprogramming T-cell metabolism constitute a viable approach to enhance the efficacy of TCR and CAR therapy in solid tumors [195].

#### Lipid supplementation

Furthermore, lipid metabolism is critical for T cell function, especially in the TME, where T cells compete with malignant cells for nutrients [ 178, 196, 197] . Lipid metabolic remodeling has also been explored to delay T-cell senescence and enhance anti-tumor potency [198], with preclinical results showing improved T-cell receptor clustering and sustained activation for better anti-tumor efficacy [199]. Studies indicate that enhancing fatty acid catabolism in T cells can improve their effector functions and persistence in the tumor microenvironment, thereby increasing the efficacy of immunotherapies such as CAR-T cells [ 200, 201] . Additionally, targeting lipid metabolism in Tregs can reduce their immunosuppressive function and strengthen the anti-tumor immune response [202]. Nanoparticle-mediated lipid metabolic reprogramming shows promise for improving T cell function in the TME. For example, nanoparticles can deliver lipid-modulating agents to T cells, promoting fatty acid oxidation over glycolysis and supporting T cell survival in glucose-deficient environments while enhancing their cytotoxicity against tumors [203]. Liposomal avasimibe, which modulates cholesterol in T cells, has improved T cell receptor clustering and activation, increasing anti-tumor efficacy in melanoma and glioblastoma mouse models [ 199, 204] .

#### Overdrive of glutaminolysis metabolism

Additionally, glutaminolysis metabolic reprogramming is essential for enhancing T cell fitness and function in the TME. Glutaminolysis provides energy and biosynthetic precursors for T cell proliferation and function, but it also fuels cancer cells, leading to nutrient competition [ 205, 206] . Targeting this pathway can reduce glutamine availability for cancer cells, enhancing T cell metabolic fitness and anti-tumor efficacy [ 207, 208] . Overactive glutaminolysis can support the energy and biosynthetic needs of activated T cells. This metabolic shift is essential for maintaining T cell function and preventing exhaustion in the hostile TME of solid tumors. Increasing glutaminolysis allows T cells to compete better for nutrients in the TME, potentially overcoming metabolic barriers and improving TCR and CAR therapy efficacy. These findings align with recent findings highlighting the importance of metabolic reprogramming in T cell-based immunotherapies and suggest that targeting metabolic pathways could enhance these treatments [184].

### Augmenting memory

Enhancing T cell fitness, particularly in terms of memory and adaptability, is crucial for cancer immunotherapy [ 209, 210] . Low CAR-T cell expansion post-infusion is associated with poor survival in patients with relapsed/refractory large B-cell non-Hodgkin lymphoma [211]. Research has shown that stem-like or memory-phenotype T cells have improved persistence and tumor control compared with conventional CAR-T cells in preclinical models [212]. Manipulating T cell differentiation pathways can also increase the degree of memory differentiation of CAR-T cells. One strategy is to target co-stimulatory domain signaling pathways to increase T cell function. The incorporation of specific co-stimulatory domains into CAR-T cells also significantly enhances their anti-tumor activity. For example, CD19 CAR-T cells with B-cell co-stimulatory molecules, such as CD79A/CD40, exhibit superior anti-tumor functions compared with those with conventional domains, such as CD28 or 4-1BB. CD79A/CD40-armed CAR-T cells upregulate genes involved in T-cell activation, proliferation, and NF-κB signaling while downregulating genes linked to exhaustion and apoptosis [213]. Additionally, kinase screening and rest of the strategies are reported to promote CAR-T-cell persistence by increasing CAR-T-cell memory [ 214, 215] . MEK inhibition also improves CAR-T-cell memory by downregulating c-Fos and JunB [216]. Moreover, the overexpression of transcription factors such as Foxo1 in CAR-T cells enhances stem-like traits, mitochondrial fitness, and persistence, increasing solid tumor control [ 182, 217] . It drives memory-associated gene expression, which is crucial for long-term efficacy. Foxo1 also promotes memory precursor T cell generation and central-memory T cell differentiation [218]. CAR-T cells engineered to overexpress the canonical AP-1 factor c-Jun have increased expansion potential, increased functional capacity, diminished terminal differentiation, and improved anti-tumor potency *in vivo* [219].

Moreover, cytokine priming is vital for enhancing T cell memory and adaptability. Key cytokines such as IL-7, IL-21, and IL-15 play crucial roles in this process. IL-7 exposure during priming increases memory CD4 ^+^ T-cell generation by promoting survival [220]. Co-expressing cytokines such as IL-15 and IL-21 can prevent functional exhaustion and enhance the resilience of engineered T cells against solid tumors [ 221– 223] . In addition, emerging studies have shown that several traditional negative cytokine signaling pathways, such as the IL-10, IL-4, and TGF-beta pathways, are also reported to help CAR-T cells resist dysfunction and mediate durable clearance of solid tumors and metastases [ 224– 226] . These findings suggest that targeting cytokines could improve T cell fitness and immune responses.

### Reverse exhaustion

T-cell exhaustion refers to a dysfunctional state of T cells characterized by reduced proliferative capacity [227], diminished cytokine production [227], and impaired cytotoxicity, often observed in chronic infections and tumors [ 228, 229] . CAR-T-cell exhaustion is a major limitation in CAR-T-cell therapy for solid tumors.

#### Reversing exhaustion through epigenetic editing

Epigenetic and genetic editing offers potential solutions to rescue exhaustion. Epigenetic changes such as DNA methylation and histone modifications are crucial for gene expression and T cell function. For instance, the epigenetic regulator EZH2 can help CAR-T cells resist exhaustion, and EZH2 inhibition enhances CAR-T-cell resistance against multiple cancers [ 230– 232] . In addition, disruption of the DNA methylcytosine dioxygenase TET2 reportedly enhances CAR-T-cell expansion, persistence, and anti-tumor efficacy, promoting a central memory phenotype and tumor remission [233]. Targeting TET2 has also been shown to restrain terminal exhaustion and improve anti-tumor responses in CAR-T cells [ 234, 235] . TET2 loss can cause antigen-independent CAR-T-cell expansion, leading to tissue infiltration and genomic instability [236]. Inhibiting histone deacetylase (HDAC) has been shown to enhance memory maintenance and resistance to exhaustion in CAR-T cells [ 237, 238] . Entinostat, an HDAC inhibitor, sustains CAR expression in CAR-NK cells, increasing their cytotoxic activity against myeloma cells [239]. HDAC inhibitors also sensitize cancer cells to CAR-T-cell clearance by upregulating NOXA [240]. These studies highlight the potential of epigenetic reprogramming in improving CAR-T-cell persistence and functionality.

#### Reversing exhaustion through transcription modification

Certain transcription factors play crucial roles in T-cell function and exhaustion [241]. For example, TOX and TOX2 are key regulators, especially in chronic infections and tumors, where they induce and maintain the exhausted state [ 242– 244] . Nuclear factor of activated T cells (NFAT) is a key transcription factor that controls the T-cell exhaustion program, influencing the expression of inhibitory receptors and impairing T-cell function [ 245, 246] . Other important factors, including IRF4, BATF, and NR4A, also act downstream of TCR signaling, contributing to the expression of inhibitory receptors and impaired T-cell function [ 247, 248] . Strategies such as genetic editing of these transcription factors on CAR-T cells have also been demonstrated to result in enhanced efficacy [ 249– 251] .

#### Reversing exhaustion through checkpoint blockade

Therapies such as anti-PD-1 and anti-PD-L1 can alter epigenetic changes [252], reverse T-cell exhaustion [253], and enhance anti-tumor activity [254], particularly in solid tumors with immunosuppressive microenvironments [ 252, 255, 256] . Strategies such as CAR-T-cell therapy and dual inhibition of the PD-1 and LAG-3 pathways have shown promise in restoring T-cell function [ 253, 257] . Combination therapies that integrate checkpoint blockades with CAR-T-cell therapy are being explored to enhance the efficacy against solid tumors [ 258, 259] . Additionally, knocking out immune checkpoint-related genes such as PD-1 promotes longer survival in tumor models by reducing exhaustion phenotypes [260]. Additionally, genetic editing to engineer CAR-T cells that express a PD-1 dominant negative receptor has improved anti-tumor efficacy in solid tumors [ 261– 263] .

### Heterogeneity or loss of tumor antigen

Treatment failure and/or disease recurrence after CAR-T-cell therapy can be caused by epitope or antigen loss [ 264, 265] . In particular, the inherently heterogeneous expression pattern of antigens in solid tumors can easily cause tumor escape after targeted immunotherapy [ 266, 267] . Therefore, targeting multiple tumor-associated antigens (TAAs) is generally expected to improve the outcome of CAR-T-cell therapy in solid tumors. When constructing dual-targeting CAR-T cells, the different co-stimulation domain choices also need to be considered [177]. We propose an approach based on dual targeting, split co-stimulatory signaling, and a shared CD3ζ chain tailored to target that allows rapid and sustained anti-tumor effects to be achieved [177]. In addition, we and others reported that, compared with traditional scFv-based CARs, bispecific-human antibody VH domain-based CARs exhibited superior tumor-killing ability [ 4, 268] .

### Other strategies to enhance CAR-T-cell therapies for solid tumors

The future of TCR and CAR therapies in solid tumors lies in continuous innovation and the integration of advanced technologies and combination strategies. By addressing the unique challenges of solid tumors, these therapies have the potential to revolutionize cancer treatment and offer new hope for patients with hard-to-treat malignancies [ 269– 272] .

#### Hybrid CAR designs and dynamic control systems

Hybrid CAR designs and dynamic control systems present significant potential for improving CAR-T-cell therapy in solid tumors. Hybrid CAR designs seek to merge the strengths of different receptor components to enhance antigen recognition and T-cell activation. For example, a study introduced a hybrid CAR in which the antigen-binding domain of a first-generation CAR was combined under the control of the CD3ε promoter, forming a CAR-CD3ε fusion protein [273]. This design harnesses the endogenous TCR signaling cascade, enabling robust CAR activity while preserving the TCR’s primary antigen recognition function, potentially increasing CAR-T-cell specificity and functionality in solid tumors. In addition, dynamic control systems also offer promising avenues for enhancing CAR-T-cell therapy. These systems enable precise and adaptable regulation of CAR-T-cell activity by responding to changes in the tumor microenvironment. They can adjust CAR-T-cell activation and function on the basis of specific signals, such as alterations in antigen density or immunosuppressive factors, thereby optimizing therapeutic effects and reducing off-target effects and T-cell exhaustion [274].

#### Optogenetic and sonogenetic CARs

Optogenetic and sonogenetic CARs, which use light-sensitive proteins or ultrasensitive heat for precise T cell activity modulation, are promising platforms. They allow dynamic regulation of CAR-T-cell activation, potentially improving their fitness and persistence in the tumor microenvironment. Optogenetic and sonogenetic CARs offer advantages such as real-time control to reduce off-target effects and cytokine release syndrome. In addition, controlled CAR expression also triggers long-lasting CAR-T-cell activation and continuous destruction of cancer cells. They also enhance CAR-T-cell adaptability to the heterogeneous and immunosuppressive solid TME through condition control [ 275, 276] .

#### SynNotch receptors

SynNotch receptors offer a key advantage in enhancing CAR-T-cell specificity and persistence by integrating multiple signals before activation, potentially reducing on-target, off-tumor toxicity, which is a major concern in solid tumor therapies due to the scarcity of highly tumor-specific antigens [277]. By programming T cells to respond only to a specific antigen combination, SynNotch circuits improve therapeutic precision [278].

Recent studies have shown that SynNotch CAR circuits can enhance solid tumor recognition and promote persistent anti-tumor activity in mouse models. For example, the identification of alkaline phosphatase placental-like 2 as a tumor-specific antigen in various solid tumors, such as mesothelioma and ovarian cancer, suggests that SynNotch CAR-T cells could effectively target a broad range of solid tumors. Additionally, the use of SynNotch receptors, such as IL-12, to control local cytokine expression increases CAR-T-cell anti-tumor activity without causing systemic toxicity [ 279, 280] . Moreover, engineering T cells with SynNotch receptors to recognize and respond to angiogenic signals in the tumor microenvironment provides another way to increase CAR-T-cell efficacy [281].

#### Combinatorial strategies

To increase the effectiveness of TCR and CAR therapy in solid tumors, combination approaches such as chemotherapy, radiation, or immune checkpoint inhibitors are being explored.

##### Radiotherapy synergy

Combining radiotherapy with CAR-T-cell therapy is a promising strategy for improving CAR-T-cell efficacy in solid tumors. Radiotherapy can modulate the TME to increase CAR-T-cell infiltration and activity while inducing immunogenic cell death to increase the anti-tumor immune response [ 282, 283] . It also promotes a chemokine gradient for better CAR-T-cell tumor infiltration and increases tumor-associated antigen expression, improving CAR-T-cell activation [284]. Additionally, radiotherapy can induce systemic immune responses through abscopal effects, which may further potentiate the efficacy of CAR-T-cell therapy [285]. These synergistic effects suggest that combining radiotherapy with CAR-T-cell therapy could help overcome barriers to CAR-T-cell effectiveness in solid tumors. Future directions for CAR-T-cell therapy in solid tumors, especially when CAR-T-cell therapy is combined with radiotherapy, should focus on tackling antigenic heterogeneity and the immunosuppressive TME [286].

##### Combining therapies with immune checkpoint blockade

Combining CAR-T-cell therapy with immune checkpoint blockade is a promising approach, as checkpoint inhibitors can counteract immunosuppressive signals within the TME, enhancing T-cell persistence and function. Preclinical and clinical studies have demonstrated a synergistic effect, suggesting that this combination could lead to more durable responses in solid tumors [ 258, 287] . As discussed in section “Reversing exhaustion through checkpoint blockade” **.**

##### Other combination strategies

The integration of oncolytic virotherapy with CAR-T-cell therapy is being explored. Oncolytic viruses can selectively infect and kill tumor cells while modulating the TME to increase immune cell infiltration and activation. This approach has shown promise in preclinical models, where oncolytic viruses have been engineered to express tumor antigens or immunomodulatory molecules, thereby increasing CAR-T-cell efficacy [288]. Additionally, approaches such as vaccines [289] and biomaterials [ 290, 291] are being investigated to increase CAR-T-cell efficiency and overcome solid tumor limitations [ 292, 293] .

## Conclusion and Future Direction

The contrast between TCR precision and CAR adaptability highlights the necessity for integrative strategies in solid tumor immunotherapy. Advances in co-stimulatory engineering, metabolic reprogramming, and epigenetic modulation offer transformative potential. The future of CAR engineering is moving toward innovative approaches such as logic-gated CARs and CRISPR-enhanced signaling. Logic-gated CARs, which utilize multiple receptors to distinguish between tumor and normal cells, offer enhanced precision and reduced off-target effects. CRISPR-enhanced signaling enables precise genomic modifications, improving the functionality and persistence of CAR-T cells. These advancements hold promise for overcoming current limitations in CAR-T-cell therapies, particularly in solid tumors.

Synthetic CARs can further learn from or integrate TCR features to improve their effectiveness. TCRs exhibit high antigen recognition precision and can present endogenous peptides, expanding the antigen repertoire. By incorporating these features, synthetic CARs could achieve more nuanced antigen recognition and sustained T-cell responses. For example, integrating TCR MHC restriction for specificity with CAR flexibility in targeting non-peptide antigens may increase therapeutic precision.

TCR-CAR hybrid approaches also present significant potential. These hybrid systems combine the strengths of both receptors, enabling T cells to respond to a broader array of tumor-associated antigens. For example, leveraging the ability of the TCR to recognize a wide range of antigens processed and presented by tumor cells while utilizing the capacity of the CAR to engage non-peptide antigens and provide co-stimulatory signals could enhance therapeutic efficacy. However, strategies for optimizing pairing and signaling integration between TCR and CAR components, as well as strategies to mitigate potential drawbacks such as off-target effects or T-cell exhaustion, must be developed.

In conclusion, the future of CAR-T-cell therapy in solid tumors hinges on continuous innovations in CAR engineering, strategic integration of TCR features, and careful development of TCR-CAR hybrid approaches. Collaborative efforts between basic researchers and clinicians remain essential for translating these strategies into durable clinical responses, providing hope for patients with refractory solid tumors.
